# *Cutibacterium avidum* Bacteria in Post-Mastectomy Breast with Prior Silicone Injections, Singapore

**DOI:** 10.3201/eid3206.251717

**Published:** 2026-06

**Authors:** Surya Subramanian, Isaac Chung De Wei, Chua Hui Wen, Allen Wei-Jiat Wong

**Affiliations:** Sengkang General Hospital, Singapore (S. Subramanian, C.H. Wen, A.W.-J. Wong); Ministry of Health Holdings, Singapore (I. Chung De Wei); Duke-National University of Singapore (NUS) Medical School, Singapore (A.W-J. Wong)

**Keywords:** *Cutibacterium avidum*, bacteria, breast silicon injection, mastectomy, breast reconstruction, Singapore

## Abstract

We isolated *Cutibacterium avidum* bacteria from remanant siliconomas noted during left mastectomy with reconstruction and right breast reduction in a 48-year-old woman with prior history of silicon injections. Chronic siliconomas can support anaerobic colonization, posing infection risks relevant to reconstructive and oncologic timelines.

We report a case of *Cutibacterium avidum* bacteria isolated from breast tissue in a 48-year-old woman with a diagnosis of left breast triple-negative carcinoma and a prior history of breast augmentation by free silicone injections to bilateral breasts. Twenty years before, the patient received multiple series of non–Food and Drug Association-approved silicone injections administered to bilateral breast tissue in all anatomic planes at an unidentified center in a foreign country. She underwent left endoscopic nipple-sparing mastectomy with pedicled transverse rectus abdominis myocutaneous flap reconstruction and contralateral reduction mammoplasty with free nipple graft. Prior research from our institution details those reconstructive techniques (*1*,*2*). Intraoperatively, we discovered multiple loculated lesions of varied size and consistency during dissection, confirming a diagnosis of siliconomas (Figure). Because of the extensive presence of lesions in both breasts, we sent tissue from the siliconomas for histopathology and conducted culture analysis, including Gram stain, aerobic, anaerobic, acid-fast bacilli, and fungal cultures, which confirmed the unusual presence of *C. avidum* bacteria. We then consulted an infectious disease specialist and, thereafter, placed the patient on a regimen of oral clindamycin 300 mg twice daily for 10 days as per standard sensitivity reports. While on that antibiotic regimen, the patient recovered without any complications.

**Figure:**
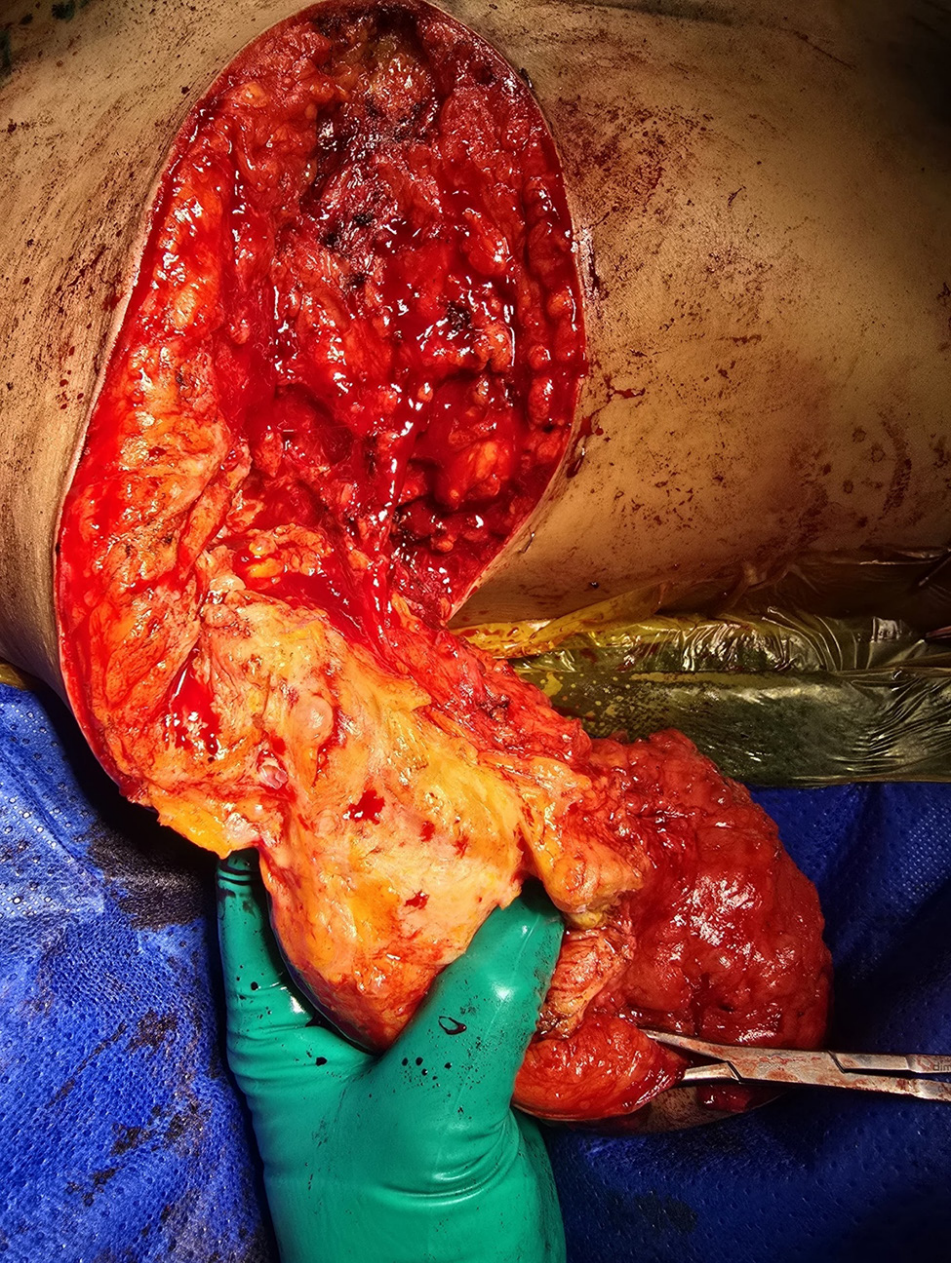
Intraoperative image from a case report of *Cutibacterium avidum* in post-mastectomy breast with prior silicone injections, Singapore. Image taken during an ongoing right breast reduction, depicting multiple loculations of foreign body embedded in the breast tissue.

*Cutibacterium* species (particularly *C. acnes*) are well recognized in breast implant infections and capsular contracture; however, our case demonstrated the colonization of this bacterial species in free silicon injection. *C. avidum* following reduction mammoplasty has been reported (*3*), and more recently has been reported in cosmetic breast implant augmentation, where the bacterium displayed true pathogenic behavior rather than acting as simply a contaminant (*4*). Other reports investigating infections relevant to aesthetic surgery have emphasized the pathogen’s association with foreign material and surgical manipulation, underscoring that *C. avidum* cannot be dismissed as a benign commensal (*5*,*6*).

Chronic siliconomas emerge as a result of a long-standing foreign-body environment. Our case suggests that siliconomas might harbor anaerobic pathogens similar to those harbored by implants or prostheses. Our findings are novel in that they suggest an extension of the clinical spectrum of *C. avidum* bacteria to silicone-injected breasts, a scenario distinct from conventional implant-based infections. Infection is an independent risk factor for flap failure (*7*,*8*), and contributes to delayed healing, flap compromise, and partial or total necrosis. Even low-grade infection can increase the risk for flap failure through inflammatory microthrombosis and impaired perfusion. Expedited healing is important in patients undergoing breast reconstruction to facilitate adjuvant chemotherapy or radiotherapy and improve overall survival (*9*). Therefore, *C. avidum* bacteria isolated from an operative field with established pathogenic potential in implant-associated infections should be regarded with appropriate diligence and treated irrespective of the clinical symptoms.

We highlight this case to alert surgeons that *C. avidum* bacteria is clinically relevant in patients with silicone injections or foreign material in breast tissue, especially as they maneuver through the course of concomitant reconstructive procedures. Awareness may influence interpretation of intraoperative cultures, antimicrobial selection, and management of late or indolent infections in this unique subgroup. In the setting of a free flap reconstruction, it is prudent to treat *C. avidum* formally with a course of antimicrobial drugs rather than risk an infection-related flap death.
